# The Epistemic Policies of Anti-Ageing Medicines in the European Union

**DOI:** 10.1007/s10728-024-00497-9

**Published:** 2024-11-19

**Authors:** Guillermo Marín Penella

**Affiliations:** Department of Philosophy and Social Work, Faculty of Philosophy and Letters, University of the Balearic Islands, Carretera de Valldemossa Km. 7, 5, Palma de Mallorca, 07071 Spain

**Keywords:** Epistemic policies, Anti-ageing medicines, European Medicines Agency, Regulatory science, Policymaking

## Abstract

Anti-ageing medicines are products intended to extend lifespan and healthspan in humans that have a good potential use in public health policies. In the European Union, their development, production and consumption are dependent on regulatory science performed by the European Medicines Agency and its associated epistemic policies. They impose, among other things, an unfavourable burden of proof, a strict standard of proof and meta-methodological constrictions related to some theoretical issues. This results in a distribution of errors that tends to reduce false positives while increasing false negatives, leading to a set of social consequences that are generally accepted when the focus is placed on conventional medicines. However, when the same epistemic policies are applied to anti-ageing medicines, the distribution of errors is imbalanced, and undesirable outcomes like research discouragement and waiting time extensions appear. Three possible strategies that policymakers could implement to unblock the situation are presented for future reflection: the consideration of ageing as a disease, the application of methodological asymmetry and the use of biomarkers during clinical research.

## Introduction

During the last two centuries, average life expectancy has increased from less than thirty to more than seventy years [1]. Nowadays, this upward trend is still continuing, and living past one-hundred is starting to be considered normal [2–3]. One of the most promising ways of reaching these new milestones has to do with the hallmarks of ageing, which are understood as the interrelated time-dependent manifestations of alterations accompanying the ageing process. These are genomic instability, telomere attrition, epigenetic alterations, loss of proteostasis, disabled macroautophagy, deregulated nutrient-sensing, mitochondrial dysfunction, cellular senescence, stem cell exhaustion, altered intercellular communication, chronic inflammation, and dysbiosis [4–5]. If the hallmarks are accentuated, ageing will be accelerated and, conversely, if they are reduced, ageing will be decelerated, stopped, or reversed [6].

Current anti-ageing strategies are therefore based on blocking genetic pathways that lead to the aforementioned hallmarks, in order to augment not only the amount of time lived, but also the period during which a human being is in good health, without chronic diseases or disabilities [7]. When attempting to increase both lifespan and healthspan, researchers rely on two interventions: making improvements to the lifestyles and using exogenous substances. Lifestyle changes include things like caloric or protein restriction, fasting, or exercise [8–9]. The intake of substances, on the other hand, refers to two different types of elements. The first is constituted of non-pharmacological compounds like curcumin, coenzymeQ10, resveratrol, probiotics, quercetin, or nicotinamide riboside [5, 10]. The second is composed of pharmacological products with the potential to reduce the ageing process through different mechanisms such as the inhibition of the GH/IGF-1 axis and the TOR-S6K pathway, the regulation of certain sirtuin proteins and some epigenetic modulators, or the reduction of inflammation [8, 11].

This work focuses on the pharmacological strategy. Although there are several drugs with promising preliminary data on their anti-ageing properties (e.g., mononucleotide of nicotinamide or a wide array of senolytics),^1^ two stand out above the rest: metformin and rapamycin [8, 11]. Metformin is a biguanide that has been shown to target all biomarkers of ageing [12], as well as extend the lifespan of mice by 7% [13–14]. Furthermore, Bannister and colleagues observed in a retrospective study in 2014 that diabetics taking metformin had greater survival not only than diabetics on other treatments (38%), but also than the non-diabetic control group (15%) [15]. Rapamycin, on the other hand, is an immunosuppressant used to prevent organ transplant rejection that has been able to extend both median survival and lifespan in mice [16–17]. Additionally, its use has also shown good capabilities to improve certain health parameters typically lost during ageing, such as walking speed, grip strength, balance, and coordination [18].

Currently, anti-ageing pharmacology is interested in transitioning from these laboratory studies to clinical practice [19]. In this way, anti-ageing drugs could be used to reduce age-related suffering, increase the autonomy and independence of older adults, and improve their life expectancy [20]. Ultimately, these benefits at the individual level would extend to society as a whole, as the incorporation of these products into public health policies could generate a significant number of advantages, including alleviating the pressure on healthcare systems and addressing typical problems faced by ageing societies [21]. However, whether anti-ageing medicines can have a truly positive impact depends largely on how they are regulated. Consequently, the research problem presented here concerns evaluating the current state of the legislation governing them in the context of the European Union (EU). To address it, it is essential to employ a methodology in which the examination of European legislation regarding medicinal products plays a fundamental role. In addition, since the aim of the paper likewise includes a critical evaluation, we will also draw upon bibliographic sources such as philosophical studies and scientific works.

In the second section, we undertake a brief analysis of the role of science in public decision making and discuss the relevance of the concept of *epistemic policies*, understood as a combination of methodological and meta-methodological options that regulates the procedures followed by regulatory scientists. In the third section, we use this concept to characterise the procedure employed during the assessments of medicines in the European Union. They are performed by the European Medicines Agency (EMA) and impose an unfavourable burden of proof, a strict standard of proof that only allows the use of evidence situated at the top of the evidential hierarchy, and meta-methodological constrictions. In the fourth section we show how the strictness of these policies generates a distribution of errors that prioritises the reduction of false positives over the increase in false negatives, something aimed at ensuring with certainty that medicinal products consumed in the region are safe and effective. However, when we focus on anti-ageing medicines, we see that the distribution of errors is imbalanced further towards an excessive production of false negatives, which leads to undesirable consequences related to the impossibility of consumption and the discouragement of research. We finish the paper briefly discussing three strategies that policymakers could use to solve the regulatory blockage of anti-ageing medicines: the consideration of ageing as a disease, the application of methodological asymmetry and the use of biomarkers during clinical research.

## Regulatory Science and Epistemic Policies

During most of recorded history, decision-making was a task undertaken primarily by kings, theocrats, and kaisers in an authoritarian way, the scientific community having no role in it [22]. However, both democratic advancement and industrial development (e.g., nuclear energy) eventually led to an empowered population who wished to regulate new technological products that, whilst offering improvements to quality of life, also represented a hazard because of their undesirable consequences (for instance, air pollution) [22–23]. In order to maximize benefits and avoid risks during production and consumption, the use of technical information provided by experts was consolidated [24]. The kind of science that was responsible for generating this knowledge to help policymakers make decisions was named regulatory science [22, 23, 25–27].

Currently, this activity is mediated by the action of regulatory agencies at the service of countries or political communities. These agencies have a set of committees in charge of carrying out the scientific activity, clearly differentiated from the academic science in both its content and context [23]. Specifically, it has been noted that regulatory scientists are under greater time pressures and are accountable to a larger number of non-scientific watchdogs [23]. Additionally, they must carry out a greater number of knowledge synthesis and evaluation tasks, such as meta-analyses and systematic reviews, compared to academic scientists, who are more focused on original research in the form of, for example, experimental studies [23]. Thus, regulatory science generates specific knowledge products such as impact models or risk-benefit assessments [27–28].^2^ However, its manner of proceeding for obtaining them is not without restrictions, but stipulated in region-dependent laws that indicate the way committees should proceed when preparing scientific information relevant to decision-making [25, 28].

For this reason, it is helpful to introduce the concept of *epistemic policies*, which refers to the content of these legal documents that indicate the procedures the scientific community should follow in a specific regulatory process. It is articulated by combinations of two types of options: methodological and meta-methodological [30]. The meta-methodological is composed of everything that is relevant for the regulatory process, but not strictly scientific. The methodological is constituted by the standards of proof, the burden of proof, and the evidential hierarchies [30–31]. The standards of proof specify the type and level of evidence required to justify a scientific claim [31–33]. Therefore, their selection has a set of associated decisions like the evidentiary requirements (*sine-qua-non* conditions that a piece of evidence must have), the scientific methods to be used, criteria for the acceptance or rejection of data, and the level of statistical significance [33]. The burden of proof has to do with the epistemic agent that must justify the hypothesis by providing information according to the required standard of proof. At the same time, it affects the type of knowledge that is considered pertinent. Finally, the evidential hierarchy indicates the value of each type of scientific evidence obtained using different methods [32, 34].

Each epistemic policy that is implemented in a regulatory process results in a particular distribution of errors [30, 33]. Some epistemic policies augment the false negatives (a situation in which it is considered that something does not have a property that it actually does possess) and others increment the false positives (a situation in which it is erroneously considered that something has a property). The distribution of errors, following the argument of inductive risk, has associated a set of social consequences that varies from case to case [35–36]. For example, Cranor [37] has shown how the greater number of false negatives that result from strict epistemic policies in toxins regulatory science, which extend the time required to perform the assessments, can lead to the population being exposed to an unacceptable amount of potential carcinogenic substances. Epistemic policies are then directly related to the non-epistemic factors via distribution of errors, so that each epistemic policy can be useful to achieve only a specific set of regulatory goals [30–32]. For this reason, non-epistemic consequences and their accordance with the goals we want to reach should be taken into account when assessing different epistemic policies [33–34].

Now, we are going to discuss the epistemic policies that regulate the authorization of medicines in the EU. Then we will examine what the distribution of errors and social consequences of these epistemic policies are, focusing on the implications they have on anti-ageing pharmaceuticals.

## The Epistemic Policies of Medicinal Products in the European Union

In the European Union, in order for a medicine to be produced and consumed, it is necessary to first obtain authorization, for which it must be demonstrated that its benefits outweigh the risks [38]. This requires carrying out scientific assessments, which the European Commission delegates to the EMA. The agency acts through specific scientific committees (Committee for Medicinal Products for Human Use [CHMP], Committee for Orphan Medical Products [COMP]…), whose epistemic policies are mainly articulated by the Directive 2001/83/CE, the Regulation (EC) Nº726/2004, and the homogenized scientific guidelines of the International Council for Harmonisation of Technical Requirements for Pharmaceuticals for Human Use (ICH).

### Methodological Aspects

#### Standard of Proof

The standard of proof implemented by the EMA is characterised by the utilization of the best available evidence in clinical practice and therapeutic processes. Accordingly, the agency requires applicants to present different types of information [38]: physico-chemical, biological, microbiological, toxicological, pharmacological, and clinical. However, the use of all non-clinical evidence (mechanistic evidence, animal tests…) has a supportive role and is mainly employed to assure the safety of volunteers during human studies, as well as to elaborate an approximate first approach to questions such as the adequate dosage [38, 39]. The risk-benefit assessment, fundamental to obtaining the authorization, will be performed considering only the evidence coming from clinical data [38, 40], which is recognised as the sole valid form of demonstrating a medicine’s efficacy and safety [41–42].

Among clinical options, EMA prioritises the strictest methodology, that is, randomized controlled trials (RCTs) [38].^3^ RCTs are considered the gold standard even in cases in which other types of information (e.g., observational studies, real world evidence) play a role in the assessment [43]. Furthermore, the amount of information gathered is also valued. Presenting various RCTs coming from different and independent laboratories greatly enhances the application [38] and augments the possibilities of obtaining authorization. This emphasis can be seen in the fact that, from 1999 to 2014, only 36 out of 795 applications of EMA were approved without this type of evidence. Of these, all used clinical studies that were not controlled [44],^4^ with the reason for the exception being the solution of unmet medical needs (orphan conditions or life-threatening situations) or the unavailability to obtain comprehensive data about safety and efficacy under normal conditions of use due to ethical, logistical, or scientific reasons [38, 45–47]. However, the presence of these situations does not automatically guarantee the use of other methodologies. Even in the face of extreme emergencies such as the COVID-19 pandemic, RCTs had to be carried out before the marketing of vaccines designed to solve the problem was permitted [48–49].

In this context, we can easily conclude that the standard of proof required by EMA’s epistemic policies is the evidence provided by RCTs under the maxim “the more, the better”. The abandonment of this methodology is only possible in exceptional circumstances and, in any case, it would still play an important role in these scenarios, as the closest comparable clinical evidence is used as a substitute [50–53].

#### Evidential Hierarchy

The primacy of RCTs and the supportive role of other types of information impose a particular evidential hierarchy. This can be seen in the understanding that the EMA has of the medicine development process, which is explained in some of its guidelines [39]. According to these, every pharmaceutical is developed in a stepwise process that starts with the obtention of non-clinical data (mechanistic, animal…) that is to be used to estimate the starting doses in human trials and the parameters for clinical monitoring. Then, human data are acquired, performing first non-randomized clinical trials using low doses with few subjects that are gradually increased when positive outcomes are obtained. RCTs with normal exposures and big samples are conducted after this process, which are considered to finally demonstrate a medicine’s efficacy and safety.

In this vein, the EMA’s evidential hierarchies are based on the image of a pyramid, the top of which is occupied by information coming from RCTs meta-analysis and RCTs studies. Non-randomized studies along with low quality RCTs are situated in the next step. The bottom of the pyramid is made up of anecdotal and laboratory evidence, as well as expert opinions without explicit critical appraisal [54]. During risk-benefit assessment of medicines, methods situated in the higher echelons are considered more capable of establishing causal relationships and reducing biases, thus increasing their perceived value.

#### Burden of Proof

Although the risk-benefit assessment is performed by the EMA and its committees, the burden of proof is borne by pharmaceutical companies. They are in charge of presenting information in accordance with the required standard of proof in order to demonstrate quality, safety and efficacy of medicines [38, 55]. This means that most of the studies necessary to generate the data must be paid for by the applicant [56], which explains why the pharmaceutical industry sponsors 60% of the RCTs performed in the European Union [40]. The burden of proof also affects the type of knowledge that is considered pertinent during the procedure because at first, by default, a medicine will be considered dangerous and ineffective. Data obtained by companies will be aimed at demonstrating that this unfavourable attitude towards the medicine is false. In other words, the medicine is considered guilty until proven otherwise.

### Meta-Methodological Aspects

The regulatory process can have additional elements that are not purely methodological. These meta-methodological components could be characterized extensively. However, for the sake of simplicity, we are going to focus only on two aspects: the implementation of ethical norms and the comprehension of central concepts. The former involves restrictions on the scientific research in its studies of living beings. The UE Directive 2010/63/EU [57] tries to reduce the pain, suffering, and distress of animals through the replacement, reduction, and refinement of their accommodation and care. It also places strong limitations on the use of wild animals, non-human primates or endangered species. For its part, the Declaration of Helsinki [58] assures the protection of life, health, dignity, integrity, right to self-determination, privacy, and confidentiality of the human research subjects, as well as emphasising the necessity of being a volunteer with total freedom.

The second aspect is related to a small number of concepts that play a role during the entire process from research to final consumption. As a main notion, highlights *medicinal product*, which is understood as a substance that is intended to treat, prevent or diagnose a disease, as well as restore, correct or modify physiological functions by exerting a pharmacological, immunological or metabolic action [38, 59]. This means that a medicine must have an *indication* to improve a condition [60]. Normally, “condition” is used as a synonym for “disease”.^5^ However, when we look for the meaning of this last term in the glossary provided by the agency [52] or on its website, we do not see any clarification. The most we can do is rely either on common sense or, in the best cases, on the International Classification of Diseases [61].

Both aspects are relevant to the regulatory process and the science performed. The first one affects either species selection in animal testing or the sample sizes in the randomized clinical trials. The second, for its part, conditions the research, the authorization, and the health problems addressed. Since a medicinal product, following the standard situation, can only be authorised for a specific disease, undesirable health-related situations must be considered as so in order to be investigated and solved. If this is not the case, obtaining the authorization turns out to be impossible.

## Distribution of Errors, Social Consequences, and Anti-Ageing Medicines

The EMA’s epistemic policies are strict. They impose an unfavourable burden of proof for medicines as well as severe meta-methodological constrictions. In addition, it holds to a methodological monism in which only the most accurate information under the maxim “the more, the better” can justify the authorization of a medicine. This is so because RCTs are recognized as the only methodology capable of drawing clear causal relationships between the intervention and the observed outcome, bias reductions or decreases in the likelihood of error [62–63]. However, it should be noted that these epistemic benefits come at the cost of demanding a lot of resources in terms of time and money, something extremely important in contexts marked by limitations of funding, dependence on commercial capacity, competitiveness, and legislative complexity [64].

Following what was said in the second section, we can connect these epistemic policies to a particular distribution of errors. On one hand, they reduce almost to zero the false positives that, in the case of medicines, happens when a medicinal product is considered safe and effective without actually being so. Due to the strictness of the requirements for justification, it is highly unlikely that a medicine pass them only by chance. On the other hand, the epistemic policies dramatically increase the number of false negatives, that is, the situation in which the product is erroneously considered unsafe or ineffective. There are many situations that explain the existence of this phenomenon, one example being the case of some small or medium-sized companies that have exceptional preliminary information for some pharmaceutical but cannot afford the development of expensive confirmatory RCTs needed obtain authorization.

The social consequences that arise from the distribution of errors of the EMA’s epistemic policies are clear. Due to the reduction of false positives, it is almost certain that any patient in the EU is taking a medicine safe and effective for their disease. However, as a result of the high number of false negatives, the amount of marketed medicines is low. This reduces treatment options and could lead, in worst cases, to diseases without pharmacological solutions. Such problems have been solved through the change of the distribution of errors, applying occasionally reductions in the standard of proof [50–53]. These very little used strategies allow an increase in the availability of medicines. However, they do so in exchange of augmenting the uncertainty related to efficacy and safety considerations [65]. In this vein, the necessity to compensate between availability and certainty will always be present.

The normal trade-off that gives priority to certainty is widely accepted for conventional medicines. Nonetheless, we will argue that this approach generates a regulatory blockage when applied to anti-ageing drugs. The reason is related to both parts of the epistemic policies: methodological and meta-methodological. As we have said, anti-ageing pharmaceuticals are medicinal products which have good preliminary data (mechanistic evidence, studies in animals…) indicating a potential to extend lifespan and healthspan [12, 66]. Furthermore, some are already authorized for the treatment of specific diseases, so they possess a very high safety profile demonstrated by the best possible evidence [67–69]. However, the methodological difficulty appears when analysing their efficacy in extending human life through a standard RCT. This has to do with the time and money needed to see effects on lifespan or overall survival (according to some estimations [70], between 20 and 50 years), inasmuch as it would be necessary to wait for the deaths of both the control group and the intervention one. The meta-methodological hardness, for its part, has to do with the fact that the EMA does not consider ageing a directly targetable condition. The response that the agency gave when asked about this question using its form was the following (EMA, personal communication, May 12, 2023):

In general, an indication is a medical condition that a medicine is used for. This can include the treatment, prevention and diagnosis of a disease.

Aging is considered a natural process rather than a disease for which specific diagnostic criteria are determined and agreed by the scientific community.

Whilst the International Classification of Diseases (ICD) has a code Ageing associated decline in intrinsic capacity (MG2A), it should be noted that these codes are used for health recording and statistics on disease in primary, secondary and tertiary care, as well as on cause of death certificates. These data and statistics support payment systems, service planning, administration of quality and safety, and health services research. However, an indication related to ageing or the MG2A code would not have an approval with an indication worded “reversal of aging” but one linked to the specific clinical parameter affected by the declining capacity, provided this parameter was recognised as a clinically meaningful outcome.

This means that no pharmacological substance could be authorized to affect ageing even in principle (this is only possible for the clinically meaningful parameters caused by it). The rationale behind the decision is the consideration of ageing as a natural process, rather than a disease, which have been to this day the point of contention between regulators and stakeholders.

Independently, these two difficulties generate an imbalance in the distribution of errors for anti-ageing medicines that increases even further the production of false negatives, so the number of helpful substances whose authorization is refused is augmented. The consideration of ageing as a non-targetable condition produces this because it denies from the outset the very possibility of carrying out the risk-benefit assessment necessary to obtain authorization, which in practice, together with the unfavourable burden of proof, means considering any functional anti-ageing medicine unsafe and ineffective.^6^ For their part, the time factor resulting from a strict standard of proof (also because of the combination with the unfavourable burden of proof) would maintain the negative consideration of any of these medicines during the long research periods needed to present the required evidence.

The consequences related to anti-ageing medicines are, therefore, different from those of conventional ones. The first and most obvious one, based on the meta-methodological aspect of the epistemic policies, is the impossibility of marketing and consuming these products (if products cannot be evaluated, they cannot be used either). This inevitably leads to the abandonment of anti-ageing drug use at both the individual and societal levels. As a result, the potential benefits outlined in the introduction (such as enhancing the autonomy and independence of older adults or addressing specific challenges faced by ageing societies) would remain unrealised. Connected to this, we can highlight the discouragement of research. Because of the unfeasibility of commercialization, few companies are willing to invest in research on something that is highly unlikely to generate economic profit, a phenomenon that is further exacerbated by the need for substantial financial outlays. This fact is empirically supported, as most pharmaceutical companies investing in anti-ageing drugs are not large firms with experience and solid financial resources, but high-risk start-ups that may encounter challenges in securing the necessary means to conduct thorough research [71].

Inevitably, this leads to a reduced production of knowledge, causing the development of this type of pharmacology to progress more slowly. Such a situation is extremely relevant, given the association between knowledge and social well-being as evidenced by historical advances in areas such as farming techniques, means of transport, or vaccines. The last consequence is the unacceptable extension of the waiting time for access to treatment. Even if the meta-methodological problem were resolved, the methodological specificities of the regulatory process and the resource estimates they generate would result in the formal authorization procedure being extremely lengthy, affecting the population that could benefit the most from these products, namely, older adults who cannot afford delays.

At this point, we can not only certify the regulatory blockage of anti-ageing medicines but also warn about the problems that the implementation of a public health policy aimed at achieving certain social objectives reliant on reducing ageing could face. To bridge the gap between acknowledging the current challenges and contemplating future solutions, although a discussion of what specific actions should be taken to unblock the situation is beyond the scope of this article, we can briefly consider, with the aim of laying the foundation for future reflection, some possible strategies that regulators could apply:

1. The consideration of ageing as a disease. This action could solve most of the meta-methodological difficulties faced by anti-ageing medicines. The idea is that, by generating an indication for this condition, pharmacologically addressing it would become feasible, thereby overcoming a significant part of the regulatory obstacle. This proposal can be supported not only for this practical reason but also for other more theoretical motivations. For instance, from a pragmatist perspective that emphasises the importance of scientific practices in shaping our understanding of biological phenomena, the mere fact that geroscience treats ageing as a disease is sufficient reason to consider it as so [72]. Additionally, defending, as the EMA does, that ageing is not a disease but a natural process, is easily criticised when we consider that many natural phenomena are regarded by the agency itself as diseases (e.g., sarcopenia). However, despite this stance being proposed in philosophy of medicine and bioethics since the end of the last century [73], there are some problematic issues that must be addressed before its final implementation, such as disease mongering and ageism.
2. Methodological asymmetry.^7^ This proposal is based on the requirement for a different standard of proof to demonstrate efficacy while using evidence from RCTs for safety. A valid approach to build this proposal upon is evidential pluralism in the philosophy of science. According to this theory, both evidence of correlation and mechanistic evidence are necessary to establish causal relationships (for example, that drug X is effective in the treatment of condition Y), regardless of the methodology employed [75]. In this vein, the combination of observational studies showing correlations, along with mechanistic studies explaining the medicine’s action, could be sufficient to validate the efficacy of anti-ageing drugs. However, it is important to note that, although this approach would allow us to overcome the regulatory barriers related to the methodological aspects, it is not without trade-offs, as it could compromise the epistemic and social benefits of a policy centred on RCTs, such as greater immunity against external pressures and conflicts of interest [76–77].
3. The use of biomarkers in RCTs. Biomarkers could be thought of as biological parameters of an organism that either alone or in some multivariate composite will, in the absence of disease, better predict functional capability at some late age than will chronological age [78]. Thus, they can be used to measure the biological ageing of an individual at any given time (e.g., at the beginning of the trial and few years later) and, consequently, serve as quick indicators of anti-ageing medicines efficacy. Given that, if applied, it would no longer be necessary to wait for the deaths of the control and intervention groups, but rather simply for changes in these biological markers, their use presents excellent prospects for reducing the resources required during clinical research. However, there are some difficulties that pose challenges, making this solution not obvious. These difficulties are related not only to the lack of scientific consensus surrounding the identification of biomarkers but, especially, to the fact that biomarkers must also be validated [79]. Thus, their use in anti-ageing studies must also be accepted by the EMA, and the necessary studies for this could also require the use of resources that are unmanageable in terms of time and money [80].

In any case, we must note that the implementation of these or other legislative modifications is a difficult task that requires acting with special care. It is necessary that, while eliminating the imbalance towards false negatives to enable production, appropriate scientific requirements are also employed that keep uncertainty levels within reason. Finding the regulatory balance can result not only in advancing our knowledge about anti-ageing medicines and improving the well-being of the population, but also in fulfilling the explicit objectives of the legislation itself: the stimulation of the pharmaceutical industry and, especially, the safeguarding of public health [38].

## Conclusion

Anti-ageing medicines can extend lifespan and healthspan in humans, presenting a valuable opportunity for integration into public health policies. Like every technological product, they are regulated in a process that depends on the use of regulatory science and its associated epistemic policies. In the case of the EU, these are performed by the EMA and establish, among other things, an unfavourable burden of proof, a strict standard of proof, and meta-methodological constrictions related to some theoretical issues. The distribution of errors of these epistemic policies and their social consequences are widely accepted for the case of conventional medicines. However, due to both methodological and meta-methodological reasons, an imbalance that produces an excessive number of false negatives appears for the case of anti-ageing ones. This generates undesirable social consequences related to their use such as the impossibility of consumption or the discouragement of research. Given this situation of blockage, three possible strategies that policymakers could implement are presented for future reflection: the consideration of ageing as a disease, the application of methodological asymmetry and the use of biomarkers during clinical research.

## Footnotes

1 Our approach to the nature of supplements and medicines is pragmatist, that is, we consider something as a supplement/medicine if it is treated as so by the relevant scientific agency. However, it must be noted that the placement of mononucleotide of nicotinamide as a medicine is controversial. Most of the time, it has been treated as a supplement, and only since the end of 2022 has the FDA started to investigate it as a drug.

2 Understanding in this way the activity of regulatory scientists, we are echoing the concept of regulatory science sometimes provided by Jasanoff [25], for whom the activity is restricted only to the piece of scientific information with which the assessment is made, but not to the assessment itself. Nevertheless, our definition is more in tune with the conception held by the majority part of regulatory institutions [27] and concretely by that specifically analysed in this paper: the EMA [29].

3 In medical sciences, randomized controlled trials are scientific experiments on humans in which the volunteers are divided into two groups. One is treated with the investigated therapeutic (intervention) and the other with a placebo or a standard of care (control). Because the groups are randomized, biases are dramatically reduced, and the evidence in favour or against the investigated treatment (when statistical significance is observed) is reliable.

4 The difference between the uncontrolled studies and RCTs lies in the fact that the former does not have a control group with which the effect of the medicine is compared. Usually, it is considered that the effect is obvious or can be measured varying the doses in the treated group.

5 In very special situations, some medicines can be indicated to act on non-pathological conditions. For example, contraceptive pills are indicated to avoid unwanted pregnancies.

6 Virtually, this converts all anti-ageing medicine in a false negative.

7 The term comes from Osimani [74], who uses it to argue in favour of stricter requirements during safety assessments than during benefits one.
